# Medical Items and News of Interest to the Physician

**Published:** 1870-07

**Authors:** 


### Medical Items and News,

### Of Interest to the Profession.

CULLED FROM THE STANDARD MEDICAL JOURNALS OF THE WORLD.

NOTE.—These formulæ are intended only for the regular practitioner of medicine, and should be employed only by him, or under his immediate supervision.

---

**Napoleon's Disease.**

Nelaton calls Napoleon's malady *vesical hemorrhoids.*

**Chancre.**

M. Champonillon, a Surgeon-Major of the French Army, states that he has dressed hard or soft chancres, with finely powdered camphor, and has been very successful with it.

**Chloroform.**

Dr. Bilbroth, Professor of Surgery at the Vienna University, has witnessed 12,500 cases of chloroform narcosis, and only one death.—Another item for the *Boston Med. Jour.*

**Amputation and Chloral Hydrate.**

The *Gazette des Hopitaux* gives the report of a case in which chloral was exhibited to the extent of producing perfect anæsthesia, under which amputation of the leg was performed by M. Noir.

**Anointing.**

The French Surgeons are making considerable ado over an anointing process which they adopt in cases of atrophy, bronchitis, convulsions and diarrhœa of children. They smear the whole body with olive oil, every four or six hours.

**Carbolic Acid in Ague.**

Dr. Markey writes to the *London Lancet* that he has tried this remedy in malarial fevers, in India, without the slightest encouraging results.

---

**Prof. Liebig's Soup.**

One ounce of ox-marrow, ½ gal. of water, a sufficient quantity of vegetables—carrots, celery, cabbage, etc., boil for one hour and a quarter, then add 5 drachms of Liebig's *meat extract*, and salt q. s. This is sufficient for seven persons.—*Record.*

---

**Syphilitic Iritis.**

We have been in the habit of treating this dangerous disease in the following manner:—We give the patient half a grain of the Protoiodide of Hyd., combined (in the form of a pill) with one grain of opium, three times daily, until slight ptialism is experienced. During this time, a collyrium, composed of—

R.

Atropia sul., gr. v;
Aqua rosæ, f. ℥ j.
M.

Is applied to the eye, by means of a camel's hair pencil, twice a day.

---

**Cancer.**

Poke Root (*phytolaca decandra*) is being extensively used in the treatment of cancerous affections. It is given in the form of a tincture, (the officinal preparation) commencing with 20 drops and increasing gradually to a dessert spoonful ter die. We have administered it with considerable success for the past year.

---

**Sweating Feet.**

In the *Deutches Klinik* we find recommended a bath, made of a pound of the sulphate of zinc, to the gallon of water. The feet are bathed in this solution for twenty minutes, which prevents excessive persperation and hardens the feet, better enabling them to undergo a pedestrian tour.

---

**Ovarian Neuralgia.**

Dr. J. T. Newman, in the *Chicago Med. Examiner*, extols the use of aconite and muriate of amonia in ovarian neuralgia. His formula is as follows:

R.

Ammonæ muriatis, ʒ ij;

Tr. aconiti, f. ʒ ij;

Syrup aurantii cort., f. ʒ viij.

M.

Sig: One teaspoonful three times a day.

**Diptheria.**

The *Chicago Medical Journal* advices the following formula:

R.

Tr. ferri chloridi, ʒ ij;

Potassæ chloratis, ʒ ij;

Morphiæ muriatis, gr. ij.

M.

Sig: One teaspoonful every second or third hour. Use also with a pencil, to the throat.

---

**Severe Burns.**

Prof. Farnsworth, of the University of Iowa, states in the Cincinnati *Med. Repos.* that he has found the following prescription very useful as a local application to burns:

R.

Ol. lini,
Aq. calcis, āā f. ℥ ij;
Sat. Sol. carbolic acid, f. ʒ ij.
M.

---

**Singular Diet.**

We find a case recorded in the *Medical Times & Gazette*, of a lady who died from a tumor of the abdominal cavity, and which proved to be, at the *post mortem*, a mass of human hair which she had been in the habit of swallowing. The mass of hair weighed 4 lbs. 7 oz., was 12 inches long, 5 inches wide and nearly 4 inches in thickness. It occupied the stomach and exhibits the powerlessness of the gastric juice to dissolve hair.

---

**A New Treatment for Typhoid Fever.**

Dr. C. W. Larison, of Ringoes, N. J., in the *Trans. N. J. Med. Soc.*, states that the course of treatment adopted by him was, camphor and opium in the proportion of four grains of the former to one of the latter, as an anodyne, and nitro-muriatic acid, beef tea, milk punch, &c. He says that out of thirty well marked cases so treated, but one died. He is satisfied that in the early stages of this disease, a free use of the mineral acids is of very great value.

---

**Sleeplessness.**

Dr. Lincoln, of Boston, reports to the *Med. & Surg. Journal* his success in treating vigilance with hydrate of chloral. In cases of nervous prostration, the result of over-fatigue and anxiety, attended with sleeplessness, he gives 15 gr. doses on retiring at night, a full night's rest of seven or eight hours is insured, without any unpleasant results of any kind.

**Successful Case of Transfusion.**

Much interest has been excited in professional as well as general circles by a case of uterine hemorrhage, in which transfusion was successfully performed by Drs. Beatty, Colles, and McDonnell. About tén ounces of blood were taken from the husband of the lady.—*British Med. Jour.*

---

**Ice in Chloroform Accidents.**

The *Med. News & Libr.* says that Dr. Baillie, Surgeon to the Calcutta Native Hospital, thrusts a small piece of ice within the rectum of his patients who are threatened with syncope, from inhalation of too large a quantity of chloroform. He says this is much more readily accomplished than one would suppose; a little pressure with the ice being made over the sphincter causes it to relax, when the ice slips in, followed almost instantly by a prolonged inspiration, and then with natural breathing.

---

**Sæsarean Section.**

Dr. Beckman, of Berlin, has published the case of a woman, aged twenty-five, who, being in the eighth month of pregnancy, was seized with apoplectic symptoms, and died comotose. Conscious that there was no chance of saving the child unless it was extracted within five minutes of the mother's death, Dr. Beckman, having obtained leave from the husband, performed the Cæsarean section with an abcess lancet, the only instrument he had with him. The child weighed hardly four pounds, and by persevering efforts at resuscitation, which were carried on for full two hours and a half, the child breathed freely, and is now a strong boy, nearly three years of age.—*Medical Record.*

---

**Whooping Cough.**

Dr. John L. Atlee, of Penn., extols the following remedy:

℞.
Acidi hydrocyanici diluti, ♏ j;
Syrupi simplicis, f. ℥ j.
M.

Sig: A teaspoonful for a child six months of age, to be given at first, morning and evening. If no unpleasant symptoms, dizziness, or sickness result, in forty-eight hours, the dose may be given three times a day. One drop of the acid may be added to the receipt for every year of the child's age, above one year.

**Gout.**

Prof. John Hughes, of Edinburgh, prescribes as follows:

℞.

Potassæ nitratis, ʒ ss.;

Aquæ, f. ʒ vj.

M.

Sig: A tablespoonful every four hours.

---

**Anæsthesia.**

The reports of Guy's Hospital show that in the Eye department 3,483 patients were chloroformed without a single accident. The *Boston Med. Journal* is making a raid upon chloroform by publishing all the accidents which it can gather from all parts of the world, that have resulted from the administration of chloroform. Wonder whether it ever sees items like the above?

---

**Neuralgia.**

Brown-Sequard's formula consists of—

℞.

Ext. belladon., gr. $\frac{1}{6}$.

Ext. stramon., gr. 1·5.

Ext. can. ind., gr. $\frac{1}{4}$.

Ext. acoint., gr. $\frac{1}{3}$.

Ext. opii, gr. $\frac{1}{2}$.

Ext. hyoscyami., gr. $\frac{2}{3}$.

Ext. conii, gr. j.

Pulv. glycyr., q. s.

For one pill. S. Three to five a day.

---

**Pruritus Vulvæ.**

We have found the following formula an excellent one, as a local application to this troublesome disease:

℞.

Carbolic acid, ʒ j;

Water, f. ℥ vj;

Hyposulphate soda, ʒ ij;

Glycerine, ℥ ij.

M.

Sig: Apply to the diseased parts by means of a cloth, saturated with the mixture.

---

**Menorrhagia.**

Dr. Ruben, of Hamberg, uses the following prescription:

℞.
Ergotinæ, gr. xv;
Glycerinæ;
Aquæ destillatæ, āā f. ʒ ss.
M.

Dose—Fifteen minims.—*Med. & Surg. Reporter.*

**Saturrine Epilepsy.**

In the *Med. Times & Gazette*, we find two cases reported of epileptic paroxysms, caused by lead poisoning, and which were relieved by large doses of the Bromide of Potassium.

---

**Dysmenorrhœa.**

The following pill is prescribed by Prof. T. Jewett, of Bowdoin College:

℞.

Camphoræ, ℥ ij ss;

Ext. belladonnæ;

Quiniæ sulphatis, āā ℥ ss;

Pulv. acaciæ, q. s.

M.

For lxxx pills. One to be taken every four hours until relieved.—*Ther. Bull.*

---

**Chloral Hydrate.**

We have administered this new remedy extensively during the past three months, and can testify to its usefulness in almost all forms of pain, or where the use of morphia is indicated. To relieve pain, after the performance of a surgical operation, it is particularly useful; it is not followed by nausea, dizziness, constipation, or any of the disagreeable products of a full dose of morphia. The dose is anywhere from ten to sixty grains, dissolved in water. Morphia frequently has the contrary effect, from the one intended, upon many persons, producing wakefulness instead of sleep; in such instances we have found the chloral hydrate to act charmingly.

A very excellent article of Chloral Hydrate may be had of Kellogg & Converse, Druggists, of this city. We mention the fact for the reason that its scarcity renders it difficult to be had.

---

**Treatment of Nævus.**

Dr. H. Bateman writes as follows to the London *Lancet*:—"I am desirous of calling attention to the mode of treating nævus, which I have now successfully adopted for about fifteen years. It consists in the application of a tartarized antimony plaster to the surface of the tumor, and allowing it to remain until pustules, resembling small-pox, are produced, and sometimes still longer. The plaster I have used is ordinarily made by having one part of antim. tartarizatum mixed with two parts of melted empl. resinæ, and then spread upon thin leather or linen. It ought to cover the nævus completely, but need not extend beyond it. If it be detached from any cause before it has produced a sufficient amount of inflammation and pustulation, it should be renewed immediately. If there be too much inflammation, poultices and fomentations of warm water may be applied, should the skin be unusually insusceptible, or the nævus unusually thick, equal parts of tartarized antimony and resin plaster may be used. With comparatively thin nævi the former strength is amply sufficient, and sometimes a single plaster will produce inflammation and pustulation enough to effect a cure. Should the nævus be thicker than usual the stronger may be used."

---

**Ulceration of Os Uteri.**

Dr. Roe, of the Lying-in Hospital, Dublin, has been in the habit of using Carbolic acid as a local application, in cases of ulceration, of the os and cervix uteri, and has found it to yield results superior to any other topical treatment which he has tried. He has used it in cases where the whole round of other applications has been unsuccessful, and always with the most happy results. He agrees with Dr. Roberts, of Manchester, who last year drew the attention of the profession to the subject, in considering it a caustic, which, as regards its severity, may take intermediate rank between the nit. argenti and strong nit. acid, besides acting as a disinfectant, a matter of no small importance in those cases. Dr. Roe does not use it in as strong a form as Dr. Roberts, and does not consider the strong acid necessary in very superficial ulcerations. A mixture of one part of the strong acid with two of olive oil seems to answer all ordinary purposes; but in cases of very deep ulceration, the use of the strong acid may be called for. In such cases Dr. Roberts desires the acid to be liquified by the addition of a very small quantity of water. The application of the carbolic acid to the os uteri is best effected by soaking a little cotton wool in the liquid, securing it by a string, and introducing it through a speculum, the string being left depending out of the vagina, and the patient being directed to pull it away on the second day. This procedure is repeated in ordinary cases about twice every week. If it be desired to apply the acid to the cervical canal, it may readily be done, by passing in a gum elastic catheter smeared with the carbolic oil.—*Ther. Bull. Med. & Surg. Reporter.*

---

A French doctor having asserted that the asparagus plant affects the kidneys, all Paris have thrown the weed into the gutter.

